# Post-cardiac injury syndrome due to iatrogenic injury successfully managed medically: a case report

**DOI:** 10.1186/s13256-023-04010-z

**Published:** 2023-07-03

**Authors:** Raid Faraj, Hassan Dib, Mehdi Abdelali, Jamila Zarzur, Mohamed Cherti

**Affiliations:** 1 Mohammed V University, Rabat, Morocco; 2 Department of Cardiology B, Ibn Sina University Hospital Center, Rabat, Morocco; 3 Cardiology Center, Mohammed V Military Instruction Hospital, Rabat, Morocco

**Keywords:** Iatrogenic pericarditis, Distal wire perforation, Post-cardiac injury syndrome, Coronary angiography

## Abstract

**Background:**

Coronary artery perforation is a rare but serious complication of percutaneous coronary interventions, that may eventually lead to major and fatal events such as myocardial infarction, cardiac tamponade, and ultimately death. The risk of coronary artery perforation is more significant during complex procedures as chronic total occlusions but it can occur in other circumstances such as oversized stents and/or balloons, excessive post-dilatation, and the use of hydrophilic wires. Coronary artery perforation is often not recognized during the procedure and the diagnosis is frequently not made until later when the patient develops signs related to pericardial effusion. Thus, causing a delay in management and worsening the prognosis.

**Case presentation:**

We report a case of a distal coronary artery perforation secondary to using a hydrophilic guide in a young male patient of 52-year-old arab, initially presented with an ST-segment elevation myocardial infarction, complicated by pericardial effusion that was treated medically with a favorable outcome.

**Conclusions:**

This work highlights that coronary artery perforation is a complication that must be anticipated in high-risk situations and its diagnosis must be made early to allow adequate management.

## Introduction

The incidence of coronary artery perforation (CAP) after percutaneous coronary interventions (PCI) varies depending on the complexity of the procedure. It is generally 0.4% but can reach 4.1% during complex procedures [1]. The causes of coronary perforation are dependent on vessel size. For large vessel perforation, it’s often related to the use of high inflations pressures particularly when the lesions are highly calcified, the use of oversized stents and balloons, and also the use of aggressive plaque modification devices. Whereas in perforations of smaller and distal vessels, the main cause is the usage of hydrophilic guides. CAP can be identified during the procedure in the cath-lab. However, when the perforation is small and subtle, the diagnosis is often made outside the cath-lab with a time delay of more than a week. Several publications have reported CAP secondary to the use of hydrophilic guides but few cases have been managed exclusively medically with good outcomes as described in our case report.

Our paper was written according to the CARE guidelines [2].

## Case presentation

**Fig. 1:**
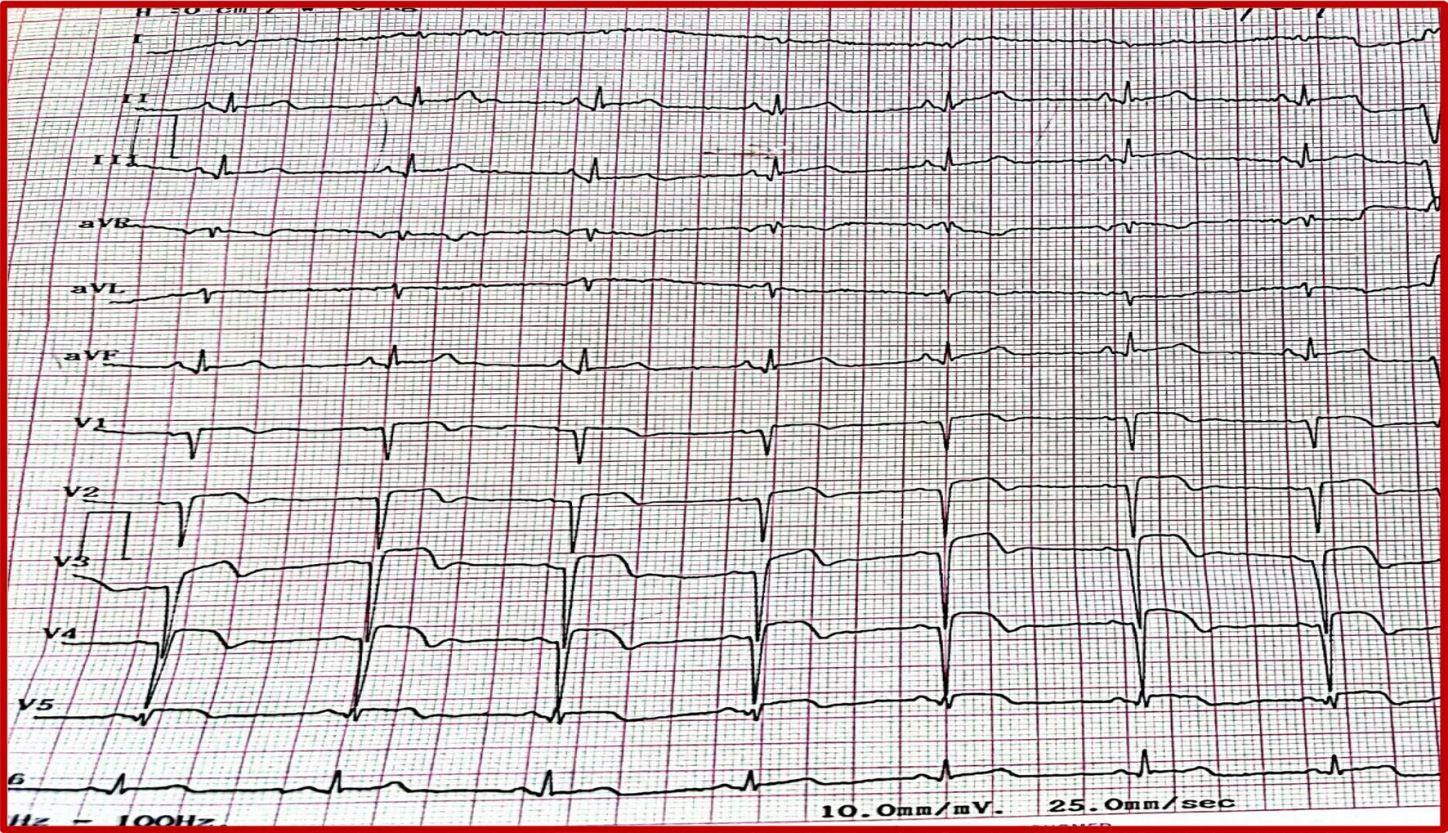
Electrocardiogram findings: ST-segment elevations in antero-septo-apical leads (V1–V4) and low lateral lead (V5) associated with necrosis-Q in antero-septo-apical leads

**Fig. 2:**
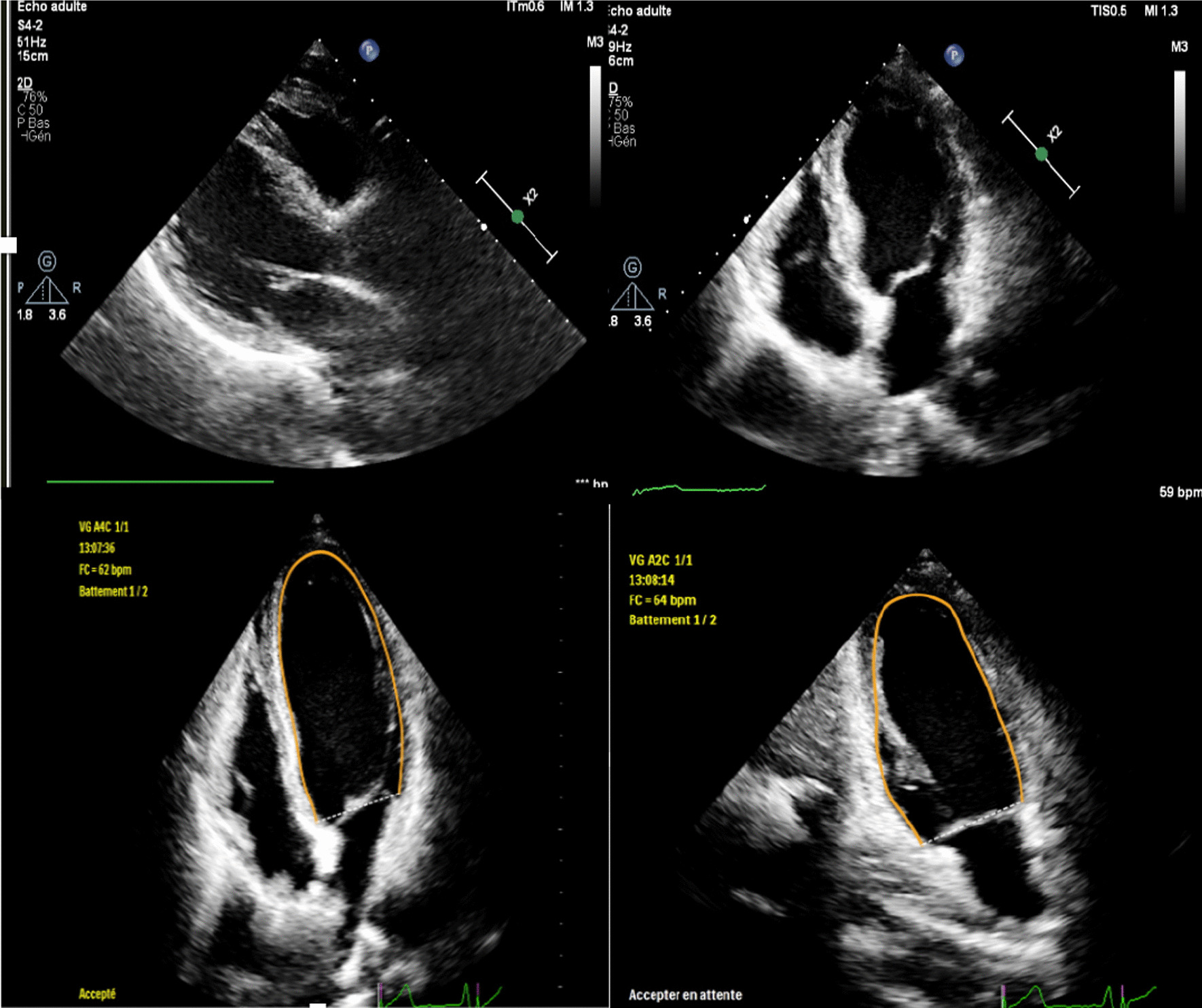
Initial transthoracic echocardiogram findings: ischemic cardiopathy aspect with moderate ventricular dysfunction. No pericardial effusion was noticed

**Fig. 3:**
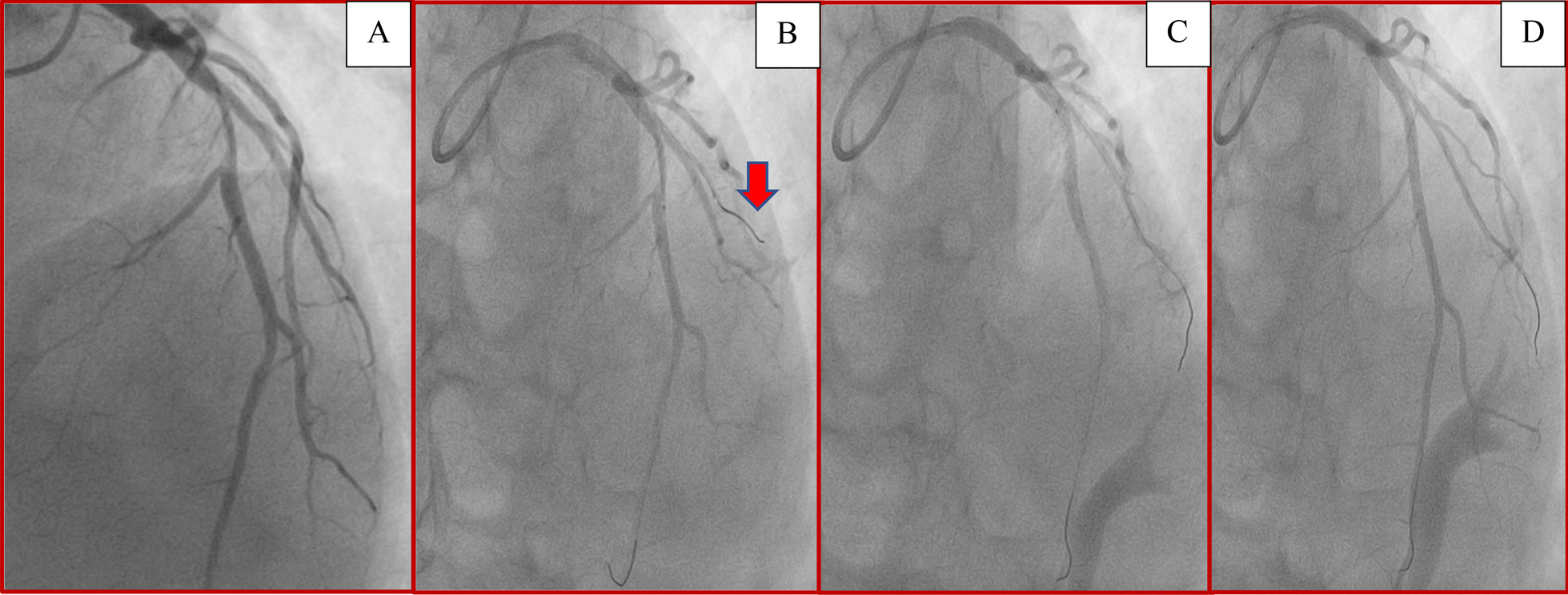
Coronary angiography findings: **A** cranial view showing a bifurcation lesion in the middle anterior interventricular artery and the diagonal branch classified as Medina (1-1-0); **B** hydrophilic guidewires insertion. Note the distal position of the diagonal branch guidewire (red arrow); **C** 2.75 × 32 mm active stent deployment followed by post-dilatation proximally with a 3.5 × 10 mm non-compliant balloon; **D** final angiographic image guides in place, showing no evidence of coronary artery perforation

**Fig. 4:**
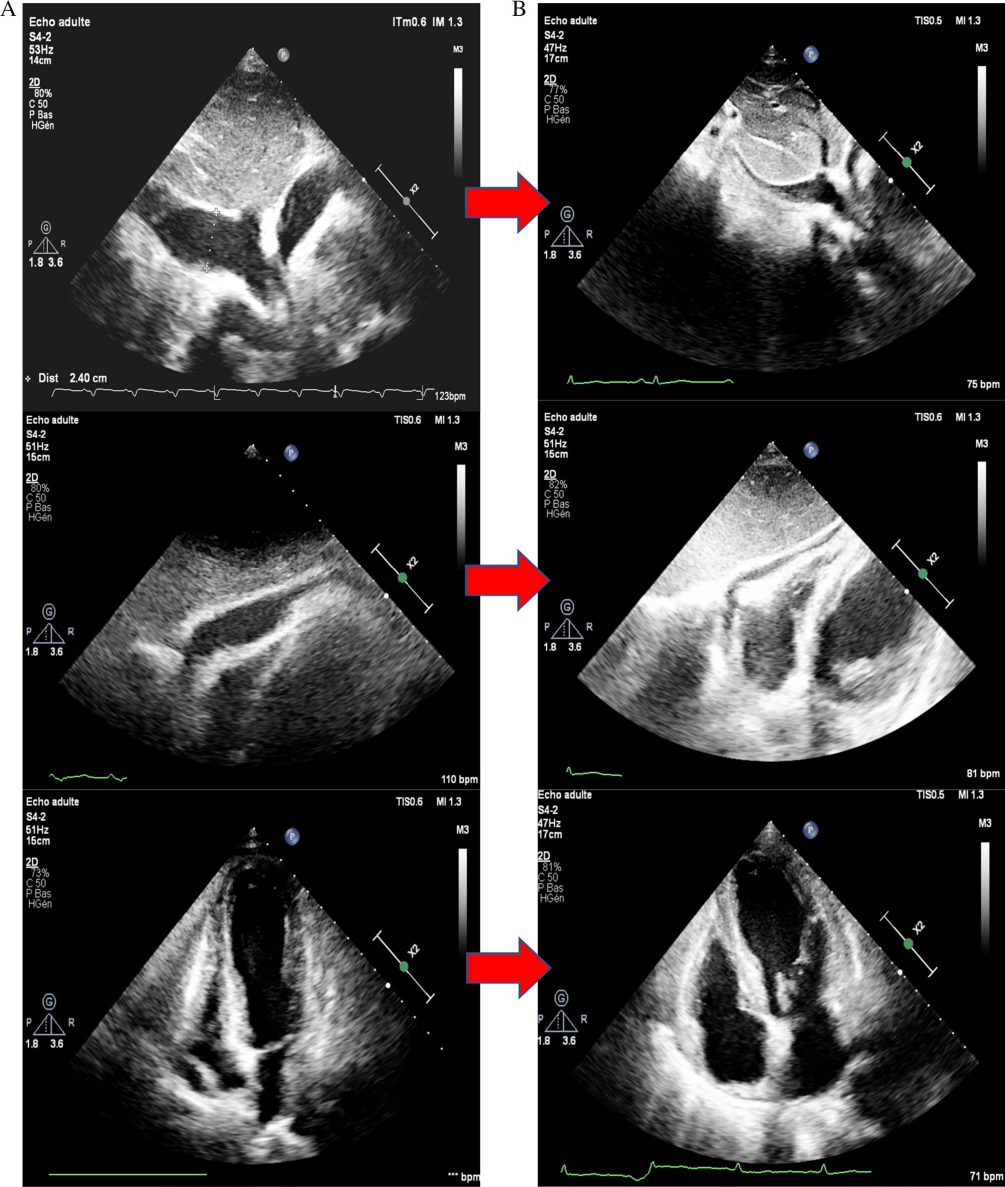
Transthoracic echocardiogram findings: **A** immediately after percutaneous coronary intervention; **B** before discharge

A 52-year-old male arab patient, a regular smoker, presented to the emergency department with acute, constructive, prolonged, intense chest pain radiating to the left upper limb, at 11 hours from the presumed onset of the pain. He did not report any particular medical or surgical history. The patient was still suffering. His blood pressure was 120/85 mmHg, his heart rate was at 91 beats per minute. Cardiac auscultation was normal. The ECG revealed ST-segment elevations in antero-septo-apical (V1–V4) and low lateral leads (V5) associated with necrosis-Q in the same leads (Fig. 1). The troponin level was as expected very high. A transthoracic echocardiogram (TTE) found an ischemic cardiopathy aspect with moderate ventricular dysfunction. We noted akinesia of the apex, septal wall, and hypokinesia of the anterior wall. No pericardial effusion was noticed (Fig. 2) Coronary angiography showed a bifurcation lesion in the middle anterior interventricular artery (AIV) and the diagonal branch classified as Medina (1-1-0). (Fig. 3A). Due to lack of means and even if they are not used in first intention, 2 hydrophilic guidewires were used. One for the AIV and another one to protect the diagonal (Fig. 3B). A 2.75 × 32 mm active stent was deployed followed by post-dilatation proximally with a 3.5 × 10 mm non-compliant balloon with a good final angiographic result but a final image without guides was not done (Fig. 3C, D). 4 hours later, the patient suddenly developed chest pain that increased with lying down. His blood pressure was 95/65 mmHg, and his heart rate was at 110 bpm. Cardiac auscultation found muffled heart sounds. The ECG did not reveal any modifications. TTE found a medium abundance pericardial effusion whose largest diameter concerned the lateral part of the right ventricle with 19 mm with an impact on the right cavities: collapse of the right atrium and the right ventricle with a dilated and not compliant lower vena cava. The respiratory variation in transvalvular blood flow velocities was normal. A significant increase in white blood cell counts and C-reactive protein (CRP) was noted. Thus, the diagnosis of post-cardiac injury syndrome (PCIS) has been established. Saline perfusion was started and he was put on an anti-inflammatory dose of Aspirin in addition to Colchicine, given the importance of the inflammatory component. Coronary angiography could not be redone due to technical problems. Close clinical and echocardiographic monitoring was conducted (every 4–6 hours initially). A marked clinical improvement with significant regression of the effusion and its impact was noted (Fig. 4). The white blood cells and the CRP became negative after 4 days. The patient was discharged and he was followed up monthly for recurrence or progression to chronic constrictive pericarditis. Fortunately, the evolution was favorable after 6 months.

## Discussion

Post-cardiac injury syndrome (PCIS) is an important cause of pericarditis, representing 21% of patients [3] It includes different subgroups, such as post-traumatic pericarditis due to iatrogenic injury as in our case. The incidence of the latter varies between 0.5 and 5% according to the trigger of the procedure [3].

In our case, it was due to the use of highly traumatic hydrophilic guidewires, particularly the one inserted in the distality of the diagonal branch. This is consistent with the literature confirming that nearly all distal vessel perforations are due to guidewire exit [4]. Fortunately, it creates a very small and self-sealing sole. The diagnosis of PCIS is established if 2 of the following 5 criteria are present: fever without alternative causes, pericarditic or pleuritic chest pain, pericardial or pleural rubs, evidence of pericardial effusion and/or pleural effusion with elevated CRP [5, 6]. Our patient’s case met 3 criteria. Pericardial effusion is a serious complication of CAP with extremely high morbi-mortality. However, short or long-term outcomes of CAP are not well described. In this regard, Jan Harnek *et al.* demonstrated through a study of 243,149 patients undergoing PCI, that the mortality in the presence of CAP could reach 16% and 52% respectively at 1 year and at 12 years [1]. It is worth noting that in almost 78% of cases, the diagnosis of PCIS was not made in the cath-lab as in our case which may delay management and further worsen the prognosis. PCI is an invasive procedure susceptible to complications that must be predicted and managed. Prevention is achieved through the right choice of guidewires with particular attention to their distal positions, especially when polymer-jacketed and stiff guidewires are used. Brilakis described an algorithm for the management of CAP [7]. The first step consists, in case of hemodynamic instability, saline perfusion with inotropic support, alerting the cardiovascular surgeons, and evaluating the necessity of pericardiocentesis. if extravasation persists, fat or coils embolization or covered stent over perforated branch origin may be considered in distal vessel perforation. In our case, a good evolution was noted after saline perfusion and the introduction of aspirin, and colchicine to treat the inflammation component, coupled with meticulous monitoring.

## Conclusion

Although rare, PCIS due to iatrogenic injury remains a delicate situation with potentially serious consequences. Thus, careful attention is required following a complex PCI or in high-risk situations. Prompt and well-planned management is essential to reduce short and long-term morbi-mortality.

## Data Availability

Data sharing is not applicable to this article as no datasets were generated or analyzed during the current study.
